# Studies on the Properties and Stability Mechanism of Double Emulsion Gels Prepared by Heat-Induced Aggregates of Egg White Protein-Oligosaccharides Glycosylation Products

**DOI:** 10.3390/foods13121822

**Published:** 2024-06-10

**Authors:** Qianwen Zhao, Cheng Lu, Cuihua Chang, Luping Gu, Junhua Li, Lulu Guo, Shende Hu, Zijian Huang, Yanjun Yang, Yujie Su

**Affiliations:** 1 State Key Laboratory of Food Science and Resources, School of Food Science and Technology, Collaborative Innovation Center of Food Safety and Quality Control in Jiangsu Province, Jiangnan University, Wuxi 214122, China; 6220111156@stu.jiangnan.edu.cn (Q.Z.); lcmjj12@163.com (C.L.); shangcuihua@163.com (C.C.); guluping@jiangnan.edu.cn (L.G.); lijunhua@jiangnan.edu.cn (J.L.); 6230112019@stu.jiangnan.edu.cn (L.G.); 6230111184@stu.jiangnan.edu.cn (S.H.); yangyj@jiangnan.edu.cn (Y.Y.); 2 College of Bioscience and Biotechnology, Hunan Agricultural University, Changsha 410128, China; 13357917857@163.com

**Keywords:** multiple emulsion, heat induced aggregate, stability, emulsion gelation, encapsulation efficiency

## Abstract

Multiple emulsions can dissolve some substances with different properties, such as hydrophilicity and lipophilicity, into different phases. They play an important role in protection, controlled release and targeted release of the encapsulated substances. However, it’s poor stability has always been one of the main problems restricting its application in the food industry. For this reason, a heat-induced aggregate (HIA) of Maillard graft product of isomalto-oligosaccharides (IMO), as well as egg white protein (EWP), was used as hydrophilic emulsifier to improve the stability of W_1_/O/W_2_ emulsions. Moreover, gelatin was added into the internal aqueous phase (W_1_) to construct W_1_/O/W_2_ emulsion-gels system. The encapsulation efficiency of HIA-stabilized W_1_/O/W_2_ emulsions remained nearly unaltered, dropping by only 0.86%, significantly outperforming the conjugates and physical mixture of IMO and EWP in terms of encapsulation stability. The emulsion-gels system was constructed by adding 5% gelatin in the W_1_, and had the highest EE% and good salt and heat stability after 30 days of storage. This experiment provides guidance for improving the stability of W_1_/O/W_2_ emulsions system and its application in the package delivery of functional substances in the food field.

## 1. Introduction

Water-in-oil-in-water (W_1_/O/W_2_) emulsions consist of three mutually separated phases, namely internal water phase (W_1_), oil phase (O) and external water phase (W_2_). Each phase can respectively dissolve different active substances and prevent their interaction, such as water-soluble and oil-soluble bioactive substances. In recent years, many scholars in the fields of medicine [1,2], cosmetics and food have made continuous contributions in developing new W_1_/O/W_2_ emulsions systems and embedding bioactive substances [3,4,5,6]. However, the poor stability of W_1_/O/W_2_ emulsions limits its application. So far, different strategies have been adopted to improve its stability, including increasing the viscosity of the inner aqueous phase (W_1_) [7], the addition of various emulsifier combinations to the aqueous phases and/or oil phase [8], and the incorporation of stabilizers and biopolymers in the outer aqueous phase (W_2_) [9,10].

Emulsifiers have an important influence on the stability of W_1_/O/W_2_ emulsions. The commonly used emulsifiers include synthetic emulsifiers, such as Tween 80 and Span 20 [11,12], and natural emulsifiers, such as proteins [13,14,15], polysaccharides or their complexes [16,17,18,19]. It is worth mentioning that proteins can be used as an effective hydrophilic emulsifier. Previous studies reported that proteins, polysaccharides, or protein−polysaccharide complexes could stabilize double emulsions through the mechanisms of spatial resistance and electrostatic repulsion and contribute to better-retaining entrapped bioactive compounds in emulsion-based delivery systems [20,21]. Current research shows that protein–carbohydrate complexes or conjugates can be used as water-soluble emulsifiers to prepare W_1_/O/W_2_ emulsions successfully [22,23]. Among them, thermally induced nanoprotein particles have great potential for application as emulsifiers [24,25,26]. Our previous research showed that the graft of egg white protein and oligosaccharide had good emulsibility and stability, which could be used for the preparation of W_1_/O/W_2_ emulsions [27].

In order to further improve the long-term storage stability of the double emulsions, the colloidal substances were added to the inner water phase. The addition of colloidal substances can effectively increase the viscosity of the emulsions, form a reversible or irreversible gel structure, make the inner or outer water phase gelatinize, slow down the movement of emulsion droplets and reduce the aggregation rate of emulsion droplets, which is conducive to the long-term stability of the W_1_/O/W_2_ system [28]. It is reported that the thermal stability and ionic stability of W_1_/O/W_2_ emulsions after gel have been significantly improved [29]. Sun et al. [30] improved the stability of W_1_/O/W_2_ emulsions by dual gelation, including the gelation of the lipid phase through oleogels and the gelation of the continuous aqueous phase through hydrogels. The combination of oleogels and hydrogels could effectively improve the stability of double emulsions through the reinforcement of the oil film and the confinement of oil globules. Hu et al. [31] investigated the influence of gel polymers present in the internal aqueous phase on the performance characteristics of W_1_/O/W_2_ emulsions and their derived microencapsulated powders. The results demonstrated that the selection of appropriate gel polymers for the internal aqueous phase is an effective strategy for enhancing the encapsulation performance of W_1_/O/W_2_ emulsions. Wang et al. [32] used kappa-Carrageenan and locust bean gum (kappa-C/LBG) as gelling polymers in the internal aqueous phase to prepare W_1_/O/W_2_ double emulsions-filled alginate hydrogel beads. The results suggested that double emulsions gelled by 2.0% kappa-C/LBG could effectively resist the oil droplets’ flocculation and inner water-droplets coalescence, and delayed the release of the inner water droplets, leading to smaller changes in the microstructure and droplets size.

Therefore, in this paper, the heat-induced aggregate (HIA) of isomalto-oligosaccharide (IMO) and egg white protein (EWP) Maillard graft product was used as the hydrophilic emulsifier, and Polyglycerol ester of polyricinoleic acid (PGPR) was used as the lipophilic emulsifier to prepare the W_1_/O/W2 emulsions. Gelatin was added to the internal aqueous phase (W_1_ phase) to prepare W_1_/O/W_2_ emulsion-gels system. The characteristics of emulsions and emulsion gels and the stability mechanism of HIA on W_1_/O/W_2_ emulsions were studied. The W_1_/O/W_2_ emulsion-gels system with good long-term stability and encapsulation performance could effectively expand the application of double emulsions.

## 2. Materials and Methods

### 2.1. Materials

Commercial hen egg white powder (EWP) (Kangde Egg Industry, Nantong, China), protein content ≥ 78%. Isomalto-oligosaccharides (IMO, purity 90%, average molecular weight 567 Da) (Baiyou Bio-Technology Co., Ltd., Langfang, China). Monascus yellow (a natural colorant) (Zhongda Hengyuan Bio-Technology Stock Co., Ltd., Luohe, China). Gelatin, Tween-80 Ammonium persulfate (APS), tetramethyl ethylenediamine (TEMED) and 40% acrylamide (Sinopharm Chemical Reagents Co., Ltd., Shanghai, China). Polyglycerol ester of polyricinoleic acid (PGPR) (Shanghai Macklin Biochemical Co., Ltd., Shanghai, China). All reagents were of analytical grade.

### 2.2. Preparation of Heat-Induced Aggregates of IMO-EWP Conjugates by Glycation

IMO-EWP conjugates and heat-induced aggregates (HIA) were prepared using a two-step method according to Wang et al. [27] and Su et al. [33], with some slight modifications. IMO-EWP conjugates were prepared in the first step. IMO and EWP (1:10, *w*/*w*) were firstly mixed and dissolved in deionized water (1:6, *w*/*v*), and then the solution was adjusted to pH 7.0 with 1 M HCl or 1 M NaOH. The supernatant was collected and filtered after centrifugation at 6000× *g* for 30 min and then freeze-dried. The dehydrated IMO-EWP mixtures were then placed in a constant temperature (60 °C) and humidity (79%) chamber (LHS-80HC-I, Bule Pard Instruments, Shanghai, China) for 3 days to promote the Maillard reaction. After that, IMO–EWP conjugates were obtained and stored at −18 °C. The second step was to prepare the thermally induced aggregates. IMO-EWP solution was prepared using IMO–EWP conjugates dissolved in deionized water at a concentration of 2% (*w*/*v*). After centrifugation at 6000× *g* for 30 min, the supernatant was collected to be filtrated, and then the filtrate was adjusted to pH 7.0 with 1 M HCl or 1 M NaOH. The graft solution was heated at 90 °C for 30 min in a thermostatic water bath and then immediately cooled in an ice bath to prepare HIA. After cooling, it was stored at 4 °C for further use.

### 2.3. Characteristic Testing of HIA

#### 2.3.1. SDS-PAGE

Sodium dodecyl sulfate-polyacrylamide gel electrophoresis (SDS-PAGE) was used to verify the connection of protein components with IMO through the coupling process. An amount of 12% separation gel and 5% concentrated gel were prepared for analysis [34]. All samples were dissolved or diluted to a protein concentration of 2.0 mg/mL. Then, a constant pressure of 80 V was maintained in the stacking gel, and the voltage was adjusted to 120 V for 1 h when the samples entered the separation gel. Finally, the gel was first dyed with Coomassie Brilliant Blue R-250 for 20 min, then the dye solution was poured out and decolorized with the decolorizing solution for 2 h until the bands were clear.

#### 2.3.2. Contact Angle and Interfacial Tension of HIA

The static contact angle and oil–water interface tension of proteins were measured with a video optical contact angle measuring instrument (OCA15EC, Delphi, Germany). The method was according to Su et al. [35], with some slight modifications. The static contact angle was determined using the drop-sitting method. The HIA solution was placed in a freeze-drying machine for 12 h. After that, the sample was thoroughly grounded in a quartz mortar, and the sample powder was pressed into a thin film using a tablet press under a pressure of 25 MPa. The injector was filled with deionized water when measuring the three-phase contact angle of water–air–protein. After a large water droplet (1–2 mL) formed at the straight needle tip, which was suspended but not dropped, it came into contact with the substrate. Subsequently, the needle was gently retracted, and water droplets were left on the protein film. The contact angle was automatically calculated using an elliptical fitting model. When measuring the three-phase contact angle of oil–air–protein, the deionized water in the injector was replaced with corn oil, and the other operations were the same as above.

The suspension drop method was used to measure the oil-water interfacial tension, according to Chen et al. [14], with slight modifications. After the sample was freeze-dried, it was prepared into a 0.25% (*w*/*w*) solution and loaded into a 1 mL syringe. The colorimetric dish containing corn oil was placed on the stage, and the interfacial tension was calculated based on the shape of the largest water droplet formed before the drop.

#### 2.3.3. Interfacial Rheology of HIA

The interfacial shear rheological properties of HIA solution were quantitatively measured by a dynamic shear rheometer (Discovery HR-3, TA Instruments, Newcastle, DE, USA), according to Li et al. [36], with slight modifications. Firstly, 15 mL of HIA solution was poured into a fixed tank, with the height of the fixed ring at 10,000 μm, ensuring that the double arm ring was suspended above the solution and not submerged. Then, 10 mL of corn oil was slowly added, avoiding the rotation of the double arm rings during this process. Dynamic time scanning was carried out at an angular velocity of 1 rad/s, with a stress range of 0.01–100%. The changes in elastic modulus and energy storage modulus with time were recorded, and measurements were repeated at least twice for each sample.

### 2.4. Preparation of W_1_/O/W_2_ Emulsions

W_1_/O/W_2_ emulsions were prepared according to the method of Zhang et al. [37] with a minor modification. Firstly, the internal water phase was prepared by dissolving a certain amount of Monascus yellow in deionized water (0.1%, *w*/*v*). Then, the oil phase was prepared by mixing a certain amount of corn oil and PGPR at 25 °C for 1 h with a magnetic stirrer. After mixing the prepared water phase and oil phase at a certain volume ratio, the W/O crude emulsion was obtained by shearing for 2 min at 13,000 rpm with a high-shear mixer (Ultra Turrax T25, IKA, Staufen, Germany). Then, the coarse emulsion was homogenized three times by a high-pressure homogenizer (AH-2010, APS, Vancouver, Canada) under a pressure of 50 MPa to obtain W/O emulsion. The W/O emulsion was then mixed with the external water phase containing HIA. The mixture was sheared by a high-speed shear (Ultra Turrax T25, IKA, Staufen, Germany) at 13,000 rpm for 2 min for pre-homogenization. After that, the W_1_/O/W_2_ emulsions were homogenized twice through a high-pressure homogenizer at a pressure of 10 MPa. The optimal formula obtained based on our previous research was 6% PGPR (*w*/*w*), 35% W_1_ phase (*v*/*v*), 0.25% HIA (*w*/*w*) and 40% W_2_ phase (*v*/*v*) [33]. The hydrophilic emulsifier in the emulsion of the control group was changed to the same concentration of EWP, IMO, EWP+IMO physical mixture and IMO-EWP conjugates, respectively. The other parameters remained unchanged in the control group.

### 2.5. Preparation of W_1_/O/W_2_ Emulsion-Gels System

The W_1_/O/W_2_ emulsion-gels system was prepared according to Sapei et al. [38], with a proper modification. Firstly, the internal water phase was prepared with a certain amount of gelatin and Monascus yellow pigment. Specifically, the gelation was dissolved with deionized water to prepare 0%, 1%, 3%, 5% and 8% (*w*/*v*) solutions, respectively, and stirred at 65 °C for 20 min, and then mixed well with (1%, *w*/*w*) Monascus yellow. The W/O primary emulsion was prepared according to the method mentioned above. The prepared W/O emulsion was immediately put into 4 °C and refrigerated for 30 min for gel transformation. After that, 30 g W/O primary emulsion and 20 g external aqueous phase containing 0.25% (*w*/*w*) HIA were mixed at 65 °C for 10 min and then sheared for 2 min at 13,000 rpm by using a high-speed shear mixture to obtain W_1_/O/W_2_ crude emulsions. The prepared crude emulsions were homogenized twice by a high-pressure homogenizer under a pressure of 10 MPa, and then immediately refrigerated at 4 °C for 30 min to obtain W_1_/O/W_2_ emulsion-gels system. The prepared fresh emulsion gels were stored at 4 °C and 37 °C for 30 days, respectively.

### 2.6. Characteristic Testing of Emulsions

#### 2.6.1. Morphology Observation

The micromorphology of W_1_/O/W_2_ emulsions was observed by light microscope (Nikon E200, Nikon Inc., Tokyo, Japan) and confocal scanning laser microscopy (CLSM) (LSM 880, Carl Zeiss, Oberkochen, Germany), respectively. For light microscopy, the fresh W_1_/O/W_2_ emulsions were placed on the glass slide, covered with the cover glass slide gently, and then left it at room temperature for 10 min. After that, the sample was observed with a 40× objective lens combined with a 10× ocular lens. For confocal scanning laser microscopy, about 100 μL fresh emulsions were dyed with 10 μL 0.1% (*w*/*v*) Nile red dye solution (solvent propylene glycol) and 10 μL 0.1% (*w*/*v*) Nile blue dye solution in the dark at room temperature for 30 min, and the morphology of emulsions were observed with a 63/1.25 lens dripping with asphalt. Nile red and Nile blue fluorescent dyes were used to excite fluorescence at 488 nm with a hydrogen/hydrogen laser and 633 nm with a helium/neon laser, respectively [33].

#### 2.6.2. Particle Size and Zeta Potential Measurements

According to the method of Liu et al. [39], the particle size and dispersion coefficient of W_1_/O/W_2_ emulsions were measured in triplicate with a Malvern Mastersizer 2000S (Malvern Instruments Ltd., Malvern, UK). The Zeta potential was determined by electrophoretic light scattering (ELS) using a Nano-ZS nanosize analyzer (Zetasizer nano ZS, Malvern, UK). The refractive index (RI) of oil drops and HIA solution in W_1_/O/W_2_ emulsions were 1.47 and 1.33, respectively.

#### 2.6.3. Rheological Analysis

According to the method of Huang et al. [37] with a slight modification, the rheological behavior of W_1_/O/W_2_ emulsions was measured with a dynamic shear rheometer (Discovery HR-3, TA Instruments, Newcastle, USA), and each sample was repeated at least twice. The apparent viscosity of each sample was measured at 25 °C using a 40 mm 4° conical plate, with a shear rate range of 0.1–100 s^−1^. The dynamic shear rheological properties of emulsions were tested on a 40 mm flat plate. The linear viscoelastic region of the sample was detected in the range of frequency 1 Hz and strained from 0.01% to 100%, and then 0.5% stress was selected as the measuring condition to conduct frequency scanning in the frequency range of 0.1–100 s^−1^.

### 2.7. Encapsulation Efficiency (EE)

The encapsulation efficiency of W_1_/O/W_2_ emulsion gels was measured according to a method described previously with some slight modifications [40]. The diluent was prepared with a solution containing 25% (*v*/*w*) ethanol and 0.5% (*w*/*w*) Tween 80 with deionized water. Firstly, the W_1_/O/W_2_ emulsion gels (1.00g) were diluted to 30 mL with diluent, and vibrated for 90 s so that the Monascus yellow pigment in the external water phase could be fully extracted. After the emulsion gels were refrigerated and centrifugated at 10 °C (4000× *g*, 10 min), the bottom layer was extracted and then refrigerated and centrifugated at 10 °C (8000× *g*, 10 min). After that, the clear liquid layer was extracted again. The final obtained clear liquid was passed through 0.22 μm PES microporous membrane. Finally, the clear and transparent filter was obtained. The content of Monascus yellow pigment not embedded in the inner water phase (W_1_) could be calculated by measuring the absorbance of filtrate at 476 nm with a UV-visible spectrophotometer (UH-5300, Hitachi Corporation, Tokyo, Japan). The EE and ES were calculated according to the following formulas.

(1) $$EE\%=(C_{i}-C_{0})/C_{i}\times 100\%$$

where EE is the encapsulation efficiency of W_1_/O/W_2_ emulsion gels. C_i_ is the concentration of Monascus yellow in W_1_ phase. C_0_ is the concentration of Monascus yellow pigment in W_2_ phase recovered by centrifugation of freshly prepared emulsion gels. 

### 2.8. Impact of Environmental Stresses on Stability of Emulsion-Gels System 

#### 2.8.1. Salt Stability

0, 10, 20, 40, 80 and 100 mM NaCl were added into the inner aqueous phase (W_1_), respectively, and W_1_/O/W_2_ emulsion gels were prepared according to the same method shown in Section 2.3. The prepared emulsion gels were stored at 4 °C, and the microstructure, Zeta potential and rheological properties of emulsion gels were further analyzed.

#### 2.8.2. Heat Stability

The emulsion gels were transferred into a 10 mL transparent serum bottle, and heated at 90 °C for 30 min, and then cooled in an ice water bath immediately. The cooled emulsion gels were stored at 4 °C and its particle size and encapsulation efficiency were analyzed.

### 2.9. Statistical Analysis

Data were analyzed using statistical software (SPSS version 22, SPSS, IBM) and expressed as mean value ± standard deviation. The statistical significance among sample groups was calculated using one-way ANOVA and Tukey’s test. Three determinations of each sample were executed with the significance level set at *p* < 0.05.

## 3. Results and Discussion

### 3.1. Characteristics of HIA

#### 3.1.1. SDS-PAGE

**Figure 1:**
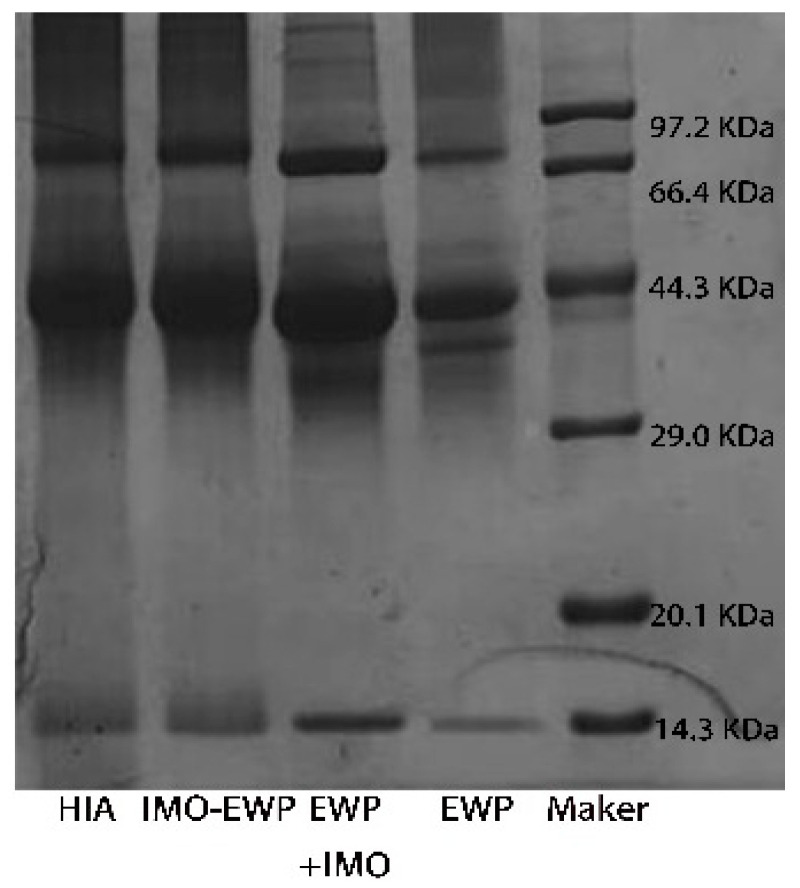
The SDS-PAGE gel electrophoresis pattern.

SDS-PAGE was used to analyze EWP, physical mixture of EWP and IMO (EWP + IMO), EWP-IMO, and HIA. As shown in Figure 1, the four main protein bands were in EWP, corresponding to ovotransferrin, ovalbumin, ovomucin and lysozyme, respectively. Compared to the EWP, the protein band position of EWP+IMO showed almost no change. The SDS-PAGE band of EWP-IMO showed a significant shift in the protein band position, indicating the generation of high molecular weight substances during the grafting process. In other words, a covalently bound graft was formed by EWP-IMO through the Maillard reaction, which increased the molecular weight of the protein. In addition, many scholars have found that bands of macromolecular substances can be observed at the boundary between concentrated and separated gels. Choi et al. [41] incubated ovalbumin and glucan at 60 °C and 79% relative humidity for 10 days and observed this phenomenon on the SDS-PAGE of the graft, indicating that the high molecular weight components of the ovalbumin and glucan graft samples were polydisperse. Jafar et al. [42] also made the same discovery when glycosylating ovalbumin and pectin. The electrophoresis bands of HIA did not show significant changes compared to IMO-EWP, indicating that heat treatment may not have altered the primary structure of the Maillard product.

#### 3.1.2. Contact Angle and Interfacial Tension

**Figure 2:**
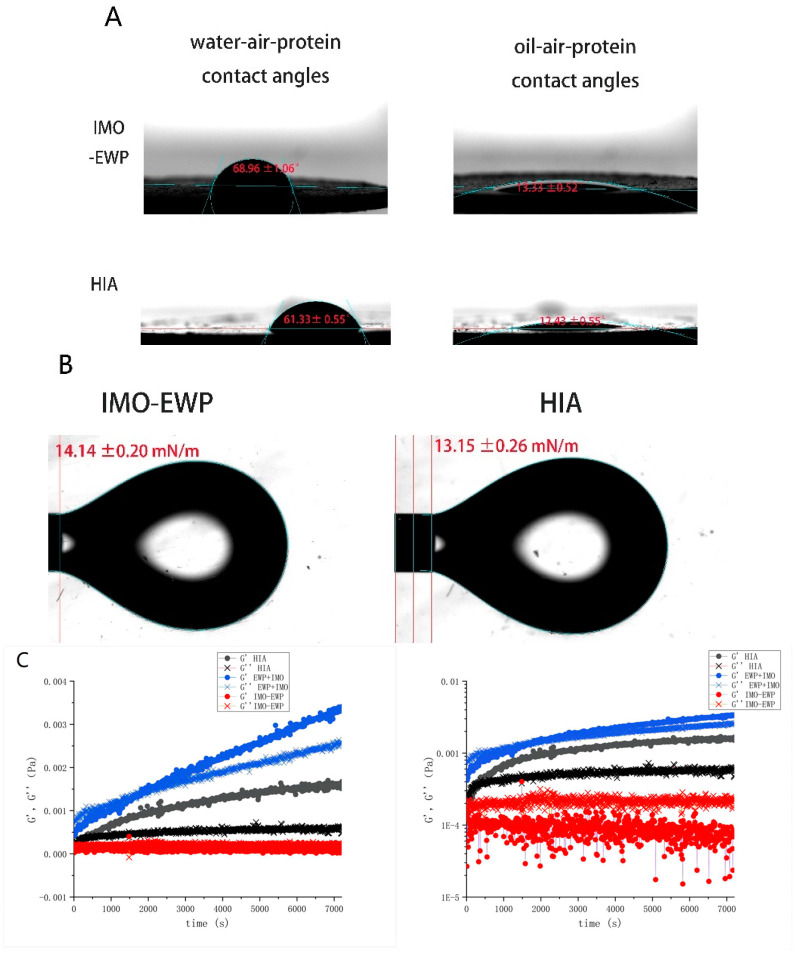
The oil–air–protein and water–air–protein contact angles (**A**), oil-water interfacial tension (**B**) and time scan curves (**C**) of IMO-EWP and HIA.

The oil–air–protein and water–air–protein contact angles of IMO-EWP and HIA were shown in Figure 2A,B. The oil–air–protein contact angle and water–air–protein contact angle of HIA were 12.43 ± 0.96°and 61.33 ± 0.55°, respectively, which confirmed the positive wettability of HIA. As is shown in Figure 2A, compared to IMO-EWP, the hydrophilicity and lipophilicity of HIA increased significantly. These results strongly supported that HIA can be used as a promising hydrophilic emulsifier to stabilize oil droplets dispersed into water phase. As shown in Figure 2B, the interfacial tension of IMO-EWP and HIA were 14.14 ± 0.20 mN/m and 13.15 ± 0.26 mN/m, respectively. The decreasing tendency of the interfacial tension was consistent with the decrease in the oil–air–protein three-phase contact angle of HIA. In addition, the liquid wetting on a solid surface was driven by the diffusion and adsorption of surfactant on the solid surface. These results reflected that HIA possessed better diffusivity and adsorption.

#### 3.1.3. Interface Rheology

The time scan curves of G′ and G″ of physical mixture of IMO and EWP (IMO+EWP), Maillard graft product (IMO-EWP) and heat-induced IMO-EWP conjugates (HIA) were shown in Figure 2C. During the test, G′ and G″ of IMO+EWP increased continuously while that of IMO-EWP and HIA tended to a stable value. This difference was related to the viscoelastic multilayer determined by unmodified EWP at the water-oil interface, which can form the network structure through hydrophobic interaction [43]. In contrast, the steric hindrance of glycated EWP was improved and more inclined to monolayer adsorption on the interface due to carbohydrates connected to the side chain. The G′ value of HIA began to exceed G″ value at about 280s, indicating that HIA formed a more elastic membrane on the interface. In addition, the G′ value of IMO-EWP was always lower than that of G″ value. One of the possible reasons is that the interfacial film formed by IMO-EWP is mainly affected by viscosity. Besides, the adsorption of Maillard graft product on the interface was too slow to form a dense interfacial film [44]. After heat treatment, the hydrophobicity of HIA was improved, which is consistent with a previous report that the 1-anilino-8-naphthalene-sulfonate (ANS) method is used to measure surface hydrophobicity [15]. Expect for this, emulsifying activity and interfacial adsorption rate of HIA were also greatly enhanced.

### 3.2. Emulsion Behavior of HIA and IMO-EWP

**Figure 3:**
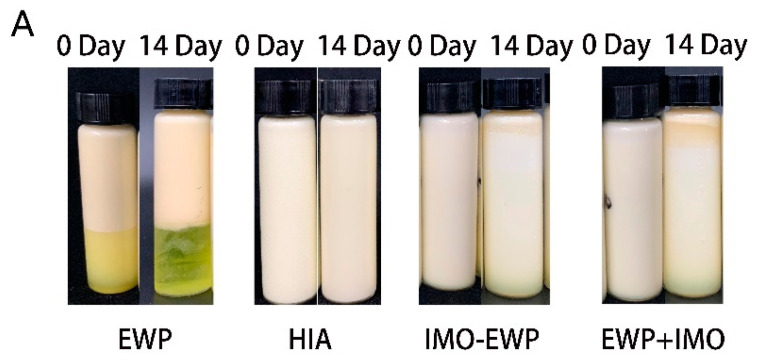

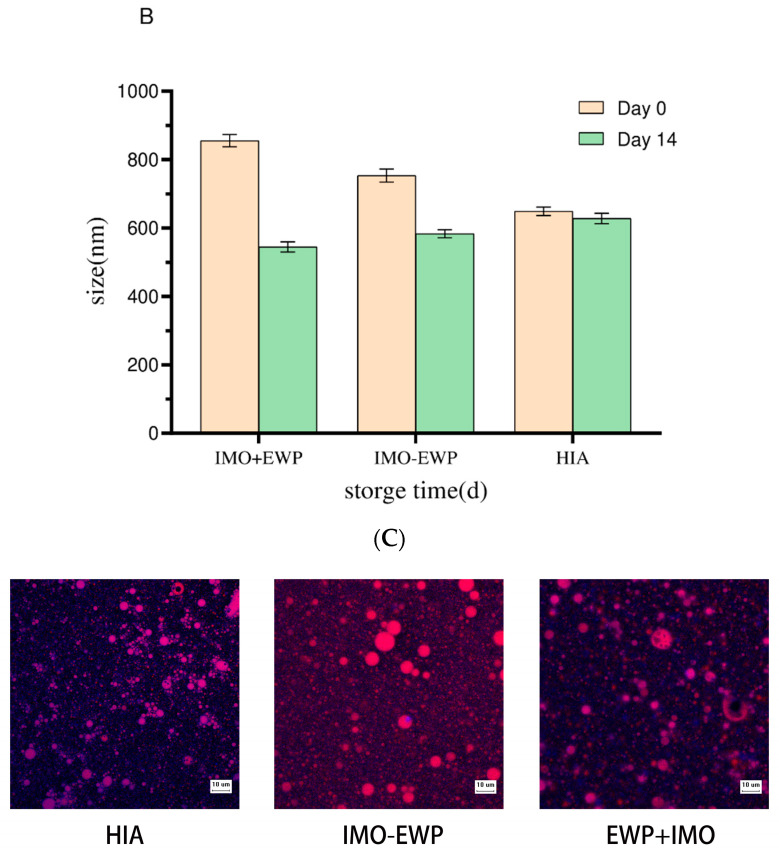
The visual appearance (**A**), mean sizes (**B**) and CLMS micrographs (**C**) of W_1_/O/W_2_ emulsions stabilized by EWP, HIA, EWP+IMO and IMO-EWP.

The changes of W_1_/O/W_2_ emulsions prepared with EWP, EWP+IMO, IMO-EWP and HIA as hydrophilic emulsifiers of external aqueous phase after 14 days storage at 4 °C were shown in Figure 3A. When EWP was used as an emulsifier, the emulsions immediately delaminated after the second homogenization, and cannot form stable W_1_/O/W_2_ emulsions, which may be related to the relatively poor emulsification of EWP. The newly prepared W_1_/O/W_2_ emulsions stabilized with EWP+IMO and IMO-EWP had slight stratification after several hours of storage, while the W_1_/O/W_2_ emulsions stabilized with HIA were relatively stable.

Moreover, the maximum particle size of the emulsions prepared by the physical mixture of IMO and EWP was about 859.53 ± 17.38 nm (Figure 3B). The confocal laser scanning microscopy images (Figure 3C) also showed that the oil droplets of the emulsions were irregular in shape, and it was difficult to observe the proteins adsorbed on the oil droplet interface. The newly prepared W_1_/O/W_2_ emulsions stabilized by HIA had the smallest particle size, which may be related to the low interfacial tension of HIA [45]. Compared with HIA, the size of oil droplets of W_1_/O/W_2_ emulsions stabilized by a physical mixture of IMO and EWP was much larger at the beginning of storage, thus leading to the instability of the emulsions.

After 14 days of storage, the particle size of the emulsions stabilized by the IMO+EWP and IMO-EWP decreased sharply, and obvious phase separation could also be observed in the appearance of the emulsions. This may be due to the instability of the droplet caused by the initial large particle size, and the continuous migration of the inner water phase to the outer water phase. The initial particle size of HIA emulsions was relatively small, and the particle size changed little during storage, so the emulsions stabilized by HIA were relatively stable.

### 3.3. Analysis of Emulsion-Gels System

#### 3.3.1. Microstructure and Appearance of W_1_/O/W_2_ Emulsion Gels

**Figure 4:**
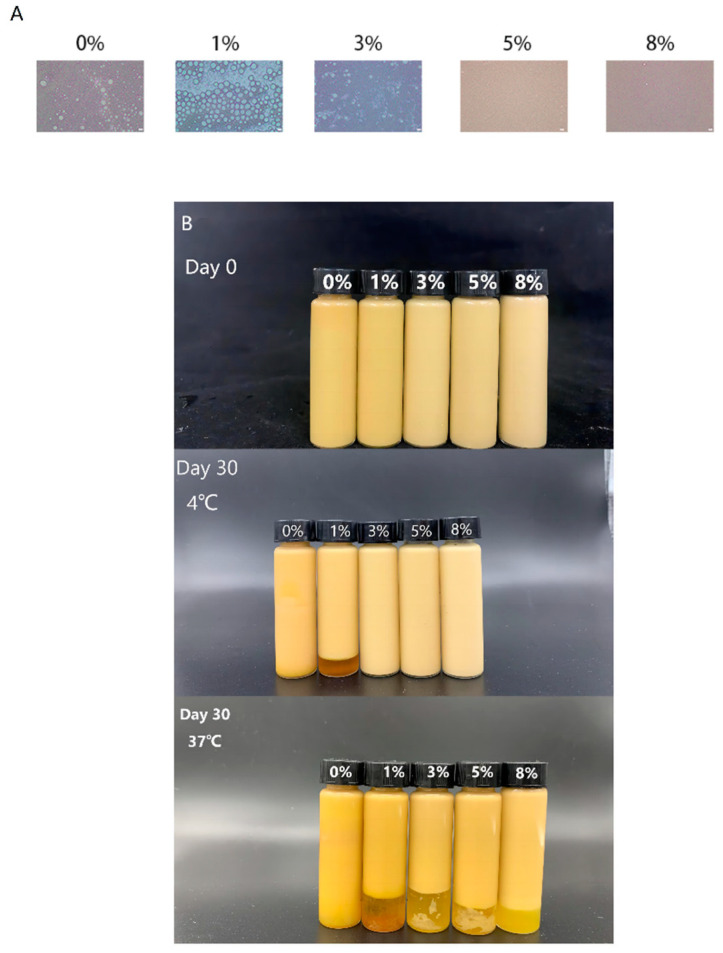
The micromorphology (**A**) and visual appearance (**B**) of W_1_/O/W_2_ emulsion gels with different NaCl content.

When the gelatin content was 1%, the size of the oil droplet was large and irregular (Figure 4A). After 30 days of storage at 4 °C, the emulsions appeared with obvious stratification, and a large amount of Monascus yellow had leaked (Figure 4B). The result showed that samples without added gelatin and with higher gelatin content exhibited more regular oil droplet morphology, while also exhibiting good stability. However, non-gel internal water droplets containing gelatin were unfavorable to the stability of W_1_/O/W_2_ emulsions. When the gelatin concentration in the internal aqueous phase of the emulsions was 1%, the non-gel internal water droplets containing gelatin were harmful to the stability of the double emulsions. The reason was that the poor ability to resist phase separation of the emulsions containing 1% gelatin in the internal aqueous phase was related to the fact that the emulsions did not form a gel at low gelatin concentration [38]. Therefore, the system containing 1% gelatin was more unstable than the system without gelatin. Zhu et al. [46] studied the influence of gelatin concentration in the internal aqueous phase on the stability of W_1_/O/W_2_ emulsions. Typically, W_1_/O/W_2_ emulsions with a gelatin content of 2% have the largest phase separation tendency. 

In addition, the confocal laser scanning microscopy images showed that gelatin and HIA (blue fluorescence) covered the inner and outer water oil interfaces, respectively. In general, the blue fluorescence increased with the increase in gelatin concentration, which indicated that higher concentrations of gelatin may form a stronger gel network and a thicker coating structure. Moreover, compared with storage at 4 °C, when the emulsions were stored at 37 °C for 30 days, all emulsions showed different degrees of phase separation.

#### 3.3.2. Particle Size and Zeta Potential Analysis

The results in Table 1 showed that the particle size of emulsions with a gelatin concentration of 8% was larger than that of 5%. The increase in the particle size of emulsions may be caused by the increased concentration of gelatin and the thicker coating structure of gelatin on the water–oil interface. This result may also be related to the increase in the viscosity of the system. The addition of gelatin led to higher viscosity of the inner water phase, making it difficult to obtain smaller particle sizes during homogenization [37].

However, when the concentration of gelatin is too low to form a stable structure of an emulsion-gels system, gelatin may compete with PGPR for adsorption, leading to an increase in particle size and instability of emulsions. With the increase of gelatin concentration, the average value of Zeta potential decreased continuously in all the samples, which may be related to the charge of gelatin.

#### 3.3.3. Rheological Properties

**Figure 5:**
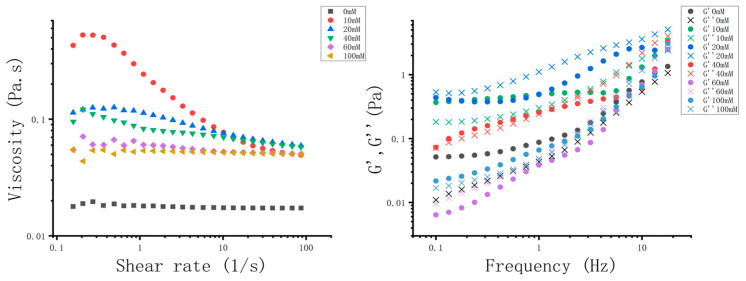
Effect of gelatin concentration on the apparent viscosity (**left**) and frequency sweep curves (**right**) of W_1_/O/W_2_ emulsions.

As shown in Figure 5, the apparent viscosity of all emulsions decreased with the increase in shear rate, which is a typical feature of shear thinning. Besides, the addition of gelatin improved the apparent viscosity of the system at all shear rates, which may be caused by two reasons. On one hand, the addition of gelatin made the emulsions more resistant to deformation [37]. On the other hand, adding gelatin may increase the interaction between droplets by promoting bridging flocculation. Furthermore, the result from the frequency sweep curve showed that, with the increase of gelatin concentration, the G‘ and G“ of the sample also increased significantly. The intersection points of G‘ and G“ shifted backward after the addition of gelatin, which indicated that a longer relaxation time was needed to form the gel structure. These results suggested that the texture of the emulsion system can be adjusted by controlling the gelatin content.

#### 3.3.4. Encapsulation Efficiency

**Figure 6:**
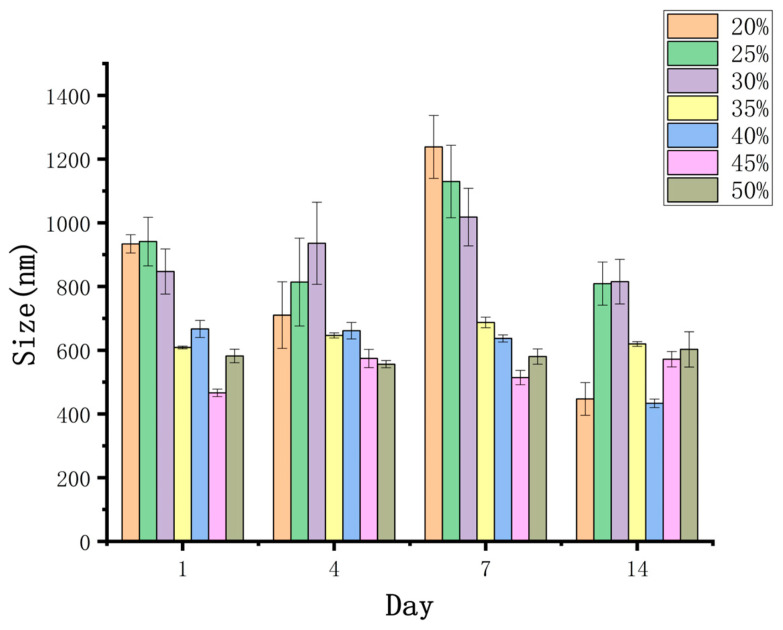
Effect of gelatin concentration on the encapsulation efficiency of Monascus yellow in W_1_/O/W_2_ emulsions after different storage periods at 4 °C.

The encapsulation efficiencies of the W_1_/O/W_2_ emulsions with different concentrations of gelation are shown in Figure 6. The results showed that all emulsions adding gelatin could contain 1% of the Monascus yellow in the internal water phase, which greatly increased the content of water-soluble substances compared with the W_1_/O/W_2_ emulsions without gelatin. As shown in Figure 5, the encapsulation efficiency of the newly prepared emulsion gels to the target substance was more than 90%. With the increase in the amount of gelatin, a thicker interfacial film can be formed to further improve the encapsulation efficiency.

With the increase in storage time, the encapsulation efficiency of emulsions without gelatin decreased significantly, which may be due to the small loading capacity of water-soluble substances in the emulsions. When the concentration of the entrapment substances increased, the Monascus yellow in the internal water phase began to migrate continuously to the external water phase. When the gelatin content was 1%, the entrapment stability of the system was the worst. After 30 days of storage, the encapsulation efficiency of Monascus yellow dropped by about 9.5%, which was related to the poor stability of the emulsions. 

On the other hand, when the addition of gelatin was 5%, the W_1_/O/W_2_ emulsion-gels system had the best encapsulation stability. After 30 days of storage at 4 °C, the encapsulation efficiency only decreased by about 1.0%. However, when further increasing the gelatin content, the encapsulation stability of the emulsion-gels system decreased. This may be because the viscosity of emulsions is too high under this condition, and it is difficult to homogenize, resulting in uneven formation of emulsions, and oil drops are more likely to break during storage.

#### 3.3.5. Salt Stability

**Figure 7:**
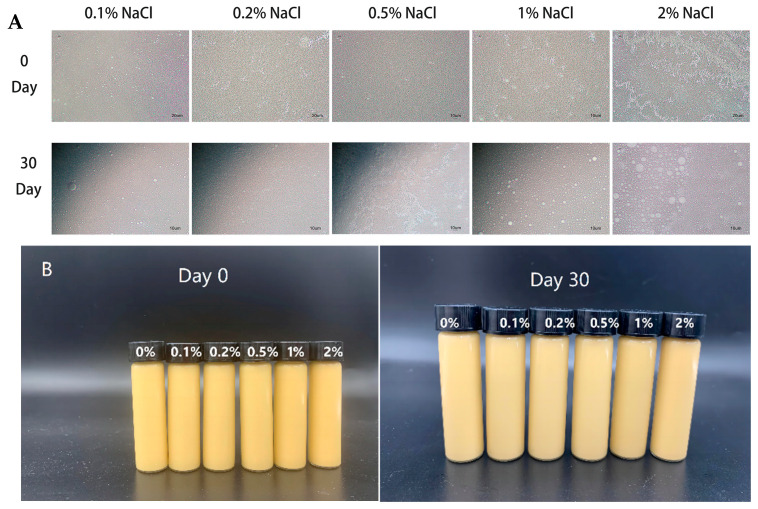
The micromorphology (**A**) and visual appearance (**B**) of W_1_/O/W_2_ emulsion gels with different NaCl content.

The results of the study on the ionic strength stability of W_1_/O/W_2_ emulsion gels are shown in Figure 7A. When adding different concentrations of NaCl into the internal water phase, the internal water phase of emulsion gels had no obvious swelling phenomenon. After 30 days of storage at 4 °C, the emulsion gels with high salt concentration remained stable (Figure 7B), but the size of oil droplets increased significantly (Figure 7A). This result was similar to the study reported by Sapei et al. [38], where gelatin and PGPR can form a dense, expandable and compressible interface layer. This interface layer can still envelop internal water droplets after expansion while delaying the release of internal aqueous substances. The high concentration of NaCl can also shield the charged sites on the gelatin polymer chain, allowing gelatin to further freely reorganize and expand which also explains that the volume of the emulsion gels containing 2% NaCl increases significantly after 30 days of storage, but the internal water phase does not expand as in our previous study [33]. This also indicated that an increase in NaCl beyond the critical concentration will lead to an increase in osmotic pressure, leading to imbalance between the internal and external water phases and the aggregation of oil droplets during storage.

#### 3.3.6. Heat Stability

**Figure 8:**
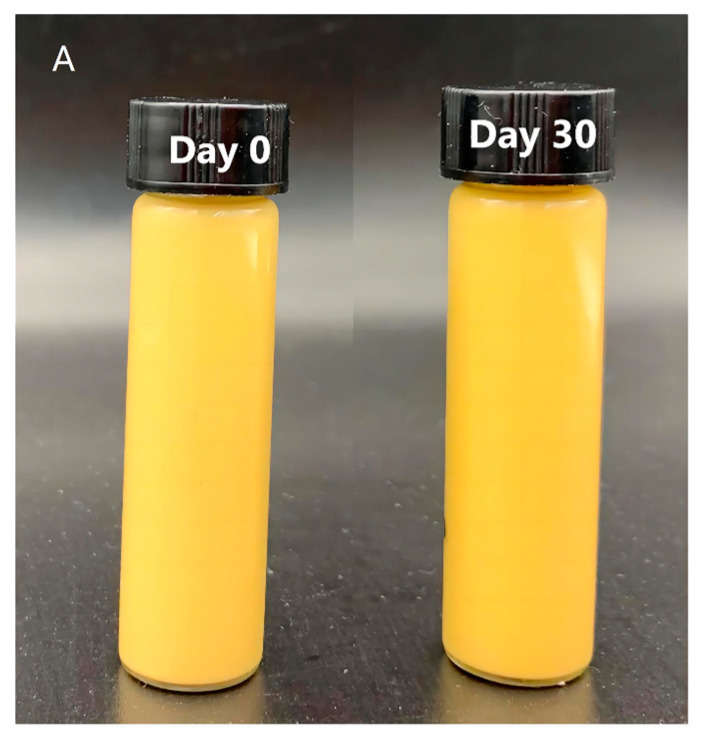

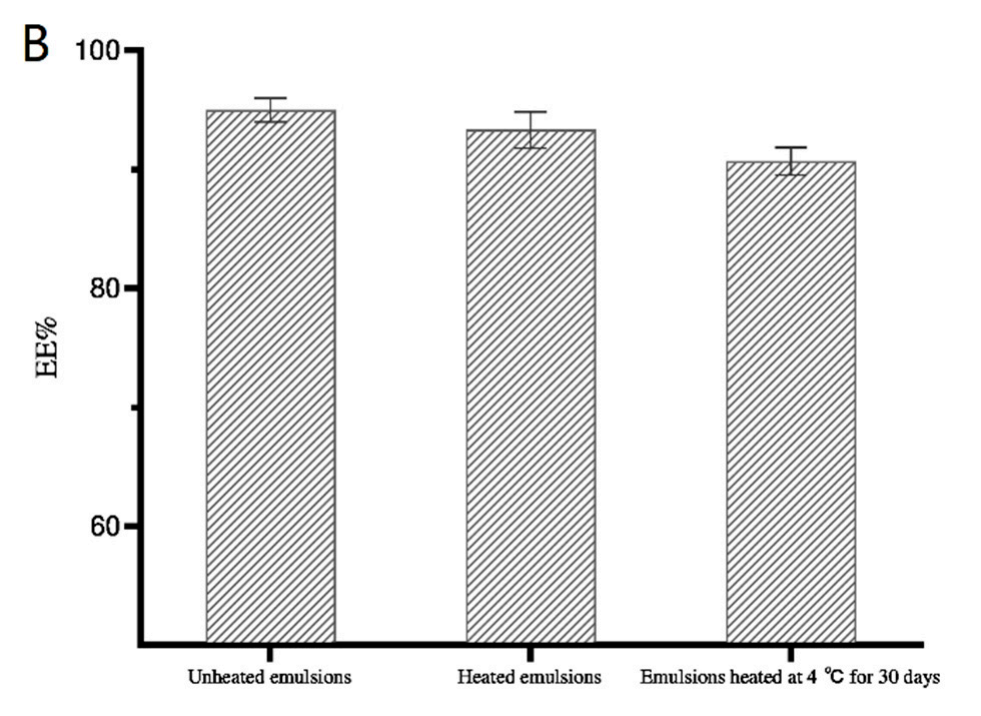
The visual appearance (**A**) and encapsulation efficiency (**B**) of W_1_/O/W_2_ emulsion gels after being heated at 90 °C for 30 min.

As shown in Figure 8A, after heat at 90 °C for 30 min, the emulsion-gels system did not delaminate, and even gel could still be formed after 30 days of storage at 4 °C. After heat treatment, the encapsulation efficiency and encapsulation stability of emulsion gels decreased slightly, but after 30 days of storage, the ES% of emulsion gels were still higher than 90% (Figure 8B). These results showed that the W_1_/O/W_2_ emulsion gels prepared in this study had good thermal stability.

## 4. Conclusions

The stability and encapsulation efficiency of W_1_/O/W_2_ emulsions stabilized by HIA were better than the emulsions stabilized by IMO+EWP and IMO-EWP. In addition, The W_1_/O/W_2_ emulsion-gels system had good ionic stability and thermal stability. The emulsion-gels system prepared by adding 0.1–2% NaCl in the internal aqueous phase showed good stability after 30 days of storage at 4 °C. The emulsion-gels system that was heated at 90 °C for 30 min and stored at 4 °C for 30 days remained stable, with an encapsulation rate of 91.2%. In general, the findings would improve the application of HIA as a hydrophilic emulsifier in multiple emulsions and expand the application of the emulsions-gels system in the delivery of bioactive substances.

## Figures and Tables

**Table 1:** Impact of gelatin concentration on mean particle diameter, polydispersity index (PDI) and zeta potential of emulsions.

Gelation%	Average Particle Size (nm)	PDI	Zeta Potential (mv)
0%	653 ± 7 ^d^	0.42 ± 0.08 ^a^	−37.83 ± 1.03 ^c^
1%	3347 ± 320 ^a^	0.29 ± 0.03 ^b^	−20.67 ± 0.12 ^b^
3%	1157 ± 18 ^b^	0.44 ± 0.02 ^a^	−19.10 ± 0.79 ^ab^
5%	805 ± 29 ^c^	0.31 ± 0.04 ^b^	−17.83 ± 0.84 ^a^
8%	960 ± 8 ^c^	0.32 ± 0.02 ^b^	−15.77 ± 0.09 ^a^

The data in the same column labeled with different letters show significant differences (*p* < 0.05).

## Data Availability

The original contributions presented in the study are included in the article, further inquiries can be directed to the corresponding author.
